# Resuscitation practices of low and normal birth weight infants in Nepal: an observational study using video camera recordings

**DOI:** 10.1080/16549716.2017.1322372

**Published:** 2017-06-02

**Authors:** Johan Wrammert, Camilla Zetterlund, Ashish KC, Uwe Ewald, Mats Målqvist

**Affiliations:** ^a^ International Maternal and Child Health, Department of Women’s and Children’s Health, Uppsala University, Uppsala, Sweden; ^b^ Health Section, UNICEF Nepal Country Office, Kathmandu, Nepal

**Keywords:** Neonatal resuscitation, low birth weight, guideline adherence, video recording, low-income population

## Abstract

**Background:** The global burden of stillbirth and neonatal deaths remains a challenge in low-income countries. Training in neonatal resuscitation can reduce intrapartum stillbirth and early neonatal mortality. Previous results demonstrate that infants who previously would have been registered as stillbirths are successfully resuscitated after such training, suggesting that there is a process of selection for resuscitation that needs to be explored.

**Objective:** To compare neonatal resuscitation of low birth weight and normal birth weight infants born at a facility in a low-income setting.

**Methods:** Motion-triggered video cameras were installed above the resuscitation tables at a maternity health facility during an intervention study (ISRCTN97846009) employing the Helping Babies Breathe resuscitation protocol in Kathmandu, Nepal. Recordings were analysed, noting crying, stimulation, ventilation, suctioning and oxygen administration during resuscitation. Birth weight, Apgar scores and sex of the infant were retrieved from matched hospital registers. The results were analysed by chi-square and logistic regression.

**Results:** A total of 2253 resuscitation cases were recorded. Low birth weight infants in need of resuscitation had higher odds of receiving ventilation (aOR 1.73, 95% CI 1.24–2.42) and lower odds of receiving suctioning (aOR 0.53, 95% CI 0.34–0.82) after adjustment for the Helping Babies Breathe intervention, sex of the infant and place of resuscitation within the facility. The rates of stimulation and administration of oxygen were the same in both groups.

**Conclusions:** Low birth weight was associated with more ventilation and less suctioning during neonatal resuscitation in a low-income setting. As ventilation is the most important intervention when the infant does not initiate breathing after birth, low birth weight was not a predictor for the decision to withhold resuscitation. Frequent routine use of suctioning of the lower airways continues to be a problem in the studied context, even after the introduction of the Helping Babies Breathe protocol.

## Background

Of the 5.8 million children who die before their fifth birthday each year, 2.9 million die during the neonatal period [1]. In addition to neonatal deaths there are approximately 2.6 million stillbirths each year, making it crucial to globally address the quality of care provided surrounding the time of birth [2]. In order to achieve the ambitious agenda of the Sustainable Development Goals, quality of care for mothers and infants around the time of birth must be strengthened [3]. An important intervention when the infant does not initiate and sustain breathing after birth is the practice of neonatal resuscitation, which can significantly reduce intrapartum-related stillbirth and neonatal mortality [4]. The Helping Babies Breathe (HBB) protocol was developed to respond to the need for increased coverage of clinical skills regarding neonatal resuscitation in low-income settings [5]. Studies on outcomes in low-income settings after training in HBB have demonstrated a reduction in intrapartum stillbirths along with reduced or unchanged early neonatal mortality [6–8]. The results imply that newborns who would have previously been classified as stillbirths were successfully resuscitated post-training. The question of whether some selection process is in play when health workers decide which infants to resuscitate in low-income settings has therefore emerged [9]. When resources are poor, for both resuscitation and postnatal care of infants, there is usually a need to prioritize. With lower gestational age and birth weight it is more probable that the birth attendant is reluctant to start resuscitation, and to instead misclassify an apnoeic live-born infant as a stillbirth [10,11]. Healthcare workers might also anticipate that withholding resuscitation could spare them from being blamed, or they might feel that they are saving the family from costs related to advanced care or future disability if the chance of survival is low [12]. The gender of the baby could also affect the decision to start resuscitation. Although male infants run a higher risk of neonatal complications, the different values placed upon sons and daughters within the social context could also constitute a factor in the selection of infants for resuscitation [13].

The clinical adherence to the steps in resuscitation protocols varies considerably and is a major challenge, especially in low-income contexts [14]. Video-recordings have been successfully used for some time to improve or evaluate health workers’ skills in resuscitation, but mostly in high-income settings [15]. Reliable and immediate interpretation of such video-recordings remains a problem that must be addressed [16]. The first step in any video analysis is to establish whether the infant in the recording was in need of resuscitation at all, or whether self-initiated breathing was sufficient. Chest movements, skin colour, muscle tone and crying can be difficult to assess from video footage, especially when the picture quality is suboptimal, or if accompanying information on heart rate and oxygen saturation is not available, as is often the case in low-income settings [10]. Apgar scores are generally available in most settings and a low Apgar score at one or five minutes clearly suggests that the infant is in need of either the initiation or continuation of resuscitation [17]. The presence of low Apgar score measurements could therefore support the data if the need for resuscitation is hard to establish from the video alone.

Premature or small-for-gestational age (SGA) infants are generally of low birth weight (LBW) and those infants clearly run a higher risk of mortality and morbidity than normal birth weight (NBW) infants [18]. LBW is defined by the World Health Organization (WHO) as a newborn with a weight below 2500 grams [19]. In low-income settings, the prevalence of premature deliveries and SGA infants is higher, and therefore a larger proportion of LBW infants are born [20]. The aim of this study was to compare neonatal resuscitation of LBW and NBW infants born at a facility in a low-income setting.

## Methods

Paropakar Maternity and Women’s Hospital (PMWH) is a tertiary government hospital in Kathmandu, Nepal, providing gynaecologic, obstetric and newborn services. In 2012 there were approximately 22,000 deliveries at the facility with a stillbirth rate of 19 and an early neonatal mortality rate of 9 per 1000 live births [8].

The STROBE (strengthening the reporting of observational studies in epidemiology) checklist was used to address the methodology used in this paper (see the Appendix). Data for this study were generated from a study of the Helping Babies Breathe (HBB) resuscitation protocol performed at the facility between 2012 and 2013 (trial registration: ISRCTN97846009) [21]. The baseline period of the study was from July to December 2012, and the intervention period from January to September 2013. It was a prospective cohort study designed to evaluate the effect of training in HBB, with outcome measures of antepartum stillbirth, intrapartum stillbirth and neonatal mortality. All women delivering at 22 gestational weeks or later were included. For the observational cohort study presented in this paper we used video data collected by cameras mounted at each resuscitation table.

Deliveries were performed at three wards: Labor Room for complicated deliveries, Newborn Centre for uncomplicated deliveries and the Operations Theatre for caesarean section. Occasionally, women delivered in the admission room. Resuscitation tables were available in each ward to allow for a swift transfer from the mother to the table in cases of apnoeic infants. Motion-triggered charge-coupled device (CCD) cameras (model MTC-505DH) were installed over the resuscitation tables located at the different wards where deliveries took place. Only the infants and the hands of healthcare providers were in the field of vision to ensure the confidentiality of the staff. No sound was recorded. The obtained videos contained time stamps and information of the location within the facility to allow them to be matched with the hospital register of the recorded delivery. If more than one delivery had the same time stamp, the place of delivery or the sex of the infant was used to distinguish them from each other. If matching was not resolved, the video observation was discarded. The video recordings were analysed by members of a surveillance team hired by the research group and the members were not affiliated to the hospital. Those surveillance officers were public health students trained in utilising a data collection tool in order to retrieve data on the initiation of resuscitation measures of stimulation, suction, ventilation and provision of oxygen. It was also noted whether the infant was crying after birth.

During the baseline period the inter-rater reliability of the analysed videos was investigated using 50 randomly selected recordings [16]. The evaluation displayed a high reliability for the use of bag and mask (98%) and suctioning (91%) but low reliability for the administration of oxygen (80%) and stimulation (78%). The inter-rater reliability of whether the infant was crying was low (56%) but the error was assumed to be uncorrelated to birth weight. Videos of non-crying infants were defined as those in need of resuscitation and thus included in this study. Because of the low reliability of the data regarding the need of resuscitation, we also compared the information on infants noted as crying or not with their Apgar scores. The cut-offs chosen for the Apgar scores were below four at one minute, and below seven at five minutes.

In the baseline period, the study protocol regarding the number of cases of infants needing resuscitation to be analysed was reviewed. The process of observing video from all the infants resuscitated turned out to be an unsustainable workload for the surveillance officers to perform. Thus, from November 2012, only videos from the resuscitation of control and case infants were analysed. In this study we therefore used video data from July to October 2012 when all infants on the resuscitation tables were analysed, and from January to September 2013 when only case and control infants were analysed, to represent the baseline and intervention period, respectively.

**Figure 1.**
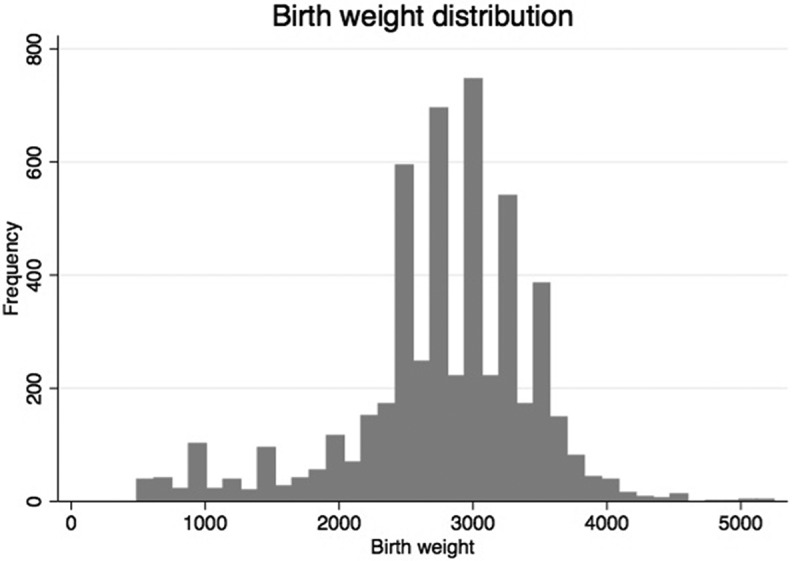
Distribution of birth weight among control and case infants born during the Helping Babies Breathe study at a maternity health facility in Kathmandu, Nepal.

LBW was defined using the WHO definition of less than 2500 grams [22]. At the facility, newborns were weighed once directly after birth using an analogue scale. Scales were validated intermittently throughout the facility during the study. As data were reviewed ahead of the statistical analysis, it was found that many infants were registered with a birth weight of exactly 2500 grams. The same pattern was seen at other multiples of 250 grams (Figure 1).

As 2500 grams represented the cut-off for LBW, this result challenged the distribution between the two groups of infants in our study. This over-registration of infants at 250- or 500-gram multiples is a known source of error for the registration of birth weights called ‘heaping’ [23]. An adjusting procedure used by the WHO recommends the re-categorisation of 25% of the infants with a weight of 2500 grams to LBW [22]. Thus, we randomly selected 25% of infants from the two study periods with a birth weight of 2500 grams and re-categorised them into the LBW group.

Pearson’s χ^2^–test was used for comparing resuscitation measures for LBW and NBW babies during baseline and intervention, respectively. Logistic regression reporting odds ratios (OR) was applied to analyse differences in resuscitation practice between LBW and NBW infants. Birth weight was set as the dependent variable and resuscitation activities as independent variables. Statistical significance was decided at *p*-values below 0.05. As the HBB intervention was expected to affect the practice of neonatal resuscitation at the facility, we first analysed each period separately and subsequently in one data-set adjusting for the intervention. As we hypothesized that gender could affect the decision to start resuscitation, this variable was adjusted for. Previous findings also indicated that the location of the table within the facility could affect the odds of receiving ventilation, thus we chose to also adjust for the place of delivery within the facility [16].

## Results

**Figure 2.**
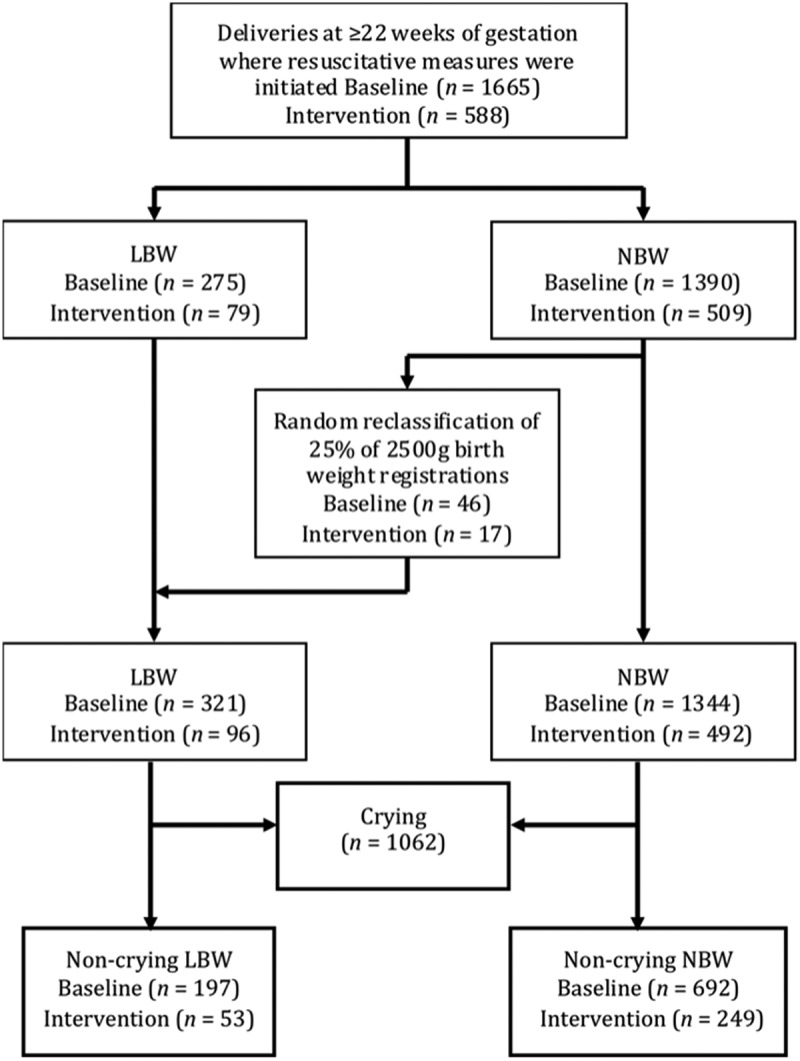
The total number of low birth weight (LBW) and normal birth weight (NBW) infants where resuscitation was recorded in the used data-set from a Helping Babies Breathe study in Kathmandu, Nepal.

During the whole study period a total of 2253 resuscitations were recorded on video. After the adjustment for heaping, 417 (19%) of those were LBW, and 1836 were NBW infants. The mean birth weights for LBW and NBW infants were 2006 and 3049 grams, respectively. Of the non-crying infants, and thus those assumed to be in need of resuscitation measures, 250 were LBW and 941 NBW infants (Figure 2). Data on gestational age were, unfortunately, missing (68%) for many infants in the baseline period. From the available data, range of gestational age among non-crying infants was 23–42 weeks in the LBW group and 33–43 weeks among the NBW infants. In the LBW group, 10 infants were born before 32 weeks of gestation. Among the NBW infants all infants were moderately premature, full-term or post-term.

The data on whether the infants were crying from the video material were crosschecked with low Apgar scores at one minute and five minutes. We found that few of the crying infants at both time intervals simultaneously had low Apgar scores, but also many of the non-crying infants did not have Apgar scores below the chosen cut-offs (Table 1).

**Table 1.** Number of crying (*n* = 1062) and non-crying (*n* = 1191) infants according to the analysis of the video recordings related to Apgar scores at one and five minutes during the Helping Babies Breathe study at a maternity facility in Kathmandu, Nepal.

	Crying *n* (%)	Non-crying *n* (%)	*p*-value
Apgar < 4 at one minute	8 (1)	145 (12)	
Apgar ≥ 4 at one minute	1054 (99)	1046 (88)	< 0.01
Apgar < 7 at five minutes	32 (3)	241 (20)	
Apgar ≥ 7 at five minutes	1030 (97)	950 (80)	< 0.01

**Figure 3.**
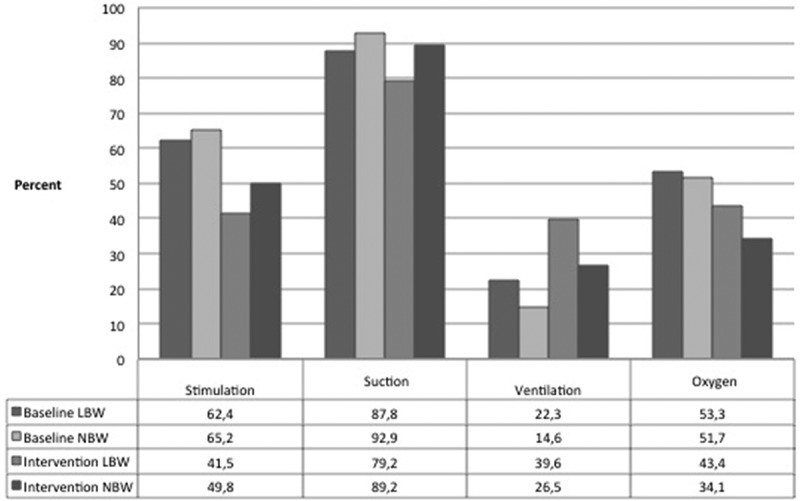
Proportion of resuscitation interventions for non-crying low birth weight (LBW) and normal birth weight (NBW) infants during the baseline and intervention study periods of the Helping Babies Breathe study at a maternity facility in Kathmandu, Nepal.

Infants from both weight groups in need of resuscitation were more frequently ventilated in the intervention period compared to baseline (*p *< 0.01). All other resuscitation interventions of stimulation, suctioning and administration of oxygen were more common during the baseline period (*p *< 0.05). Suctioning was frequently used and was performed on most non-breathing babies, both before and after the intervention (Figure 3).

Logistic regression demonstrated a difference in the interventions taken during resuscitation for the two weight groups in the baseline but not the intervention period. During baseline, odds of suctioning of LBW infants were lower (OR 0.55, 95% CI 0.33–0.92), and higher for ventilation (OR 1.68, 95% CI 1.13–2.50). Estimates did not change significantly when adjusting for gender and place of delivery. There was no difference in the odds of stimulation and administration of oxygen to LBW infants in the baseline period. During the intervention period, both weight groups were treated the same for all measures (Table 2).

**Table 2.** Odds ratios (OR) adjusted for sex and place of delivery with 95% confidence intervals for a non-crying LBW baby receiving resuscitative measures compared to a non-crying NBW baby during the baseline and intervention study periods of the Helping Babies Breathe study in Nepal.

	Baseline, *n *= 889			Intervention, *n *= 302		
	*n*	Adj. OR	*p-value*	*n*	Adj. OR	*p-value*
Stimulation						
NBW	123	Ref		124	Ref	
LBW	451	1.00 (0.71–1.40)	0.99	22	0.72 (0.39–1.33)	0.29
Suction						
NBW	643	Ref		222	Ref	
LBW	173	**0.55** (0.33–0.93)	**0.03**	42	0.47 (0.21–1.02)	0.06
Ventilation						
NBW	101	Ref		66	Ref	
LBW	44	**1.65** (1.11–2.47)	**0.01**	21	1.87 (1.00–3.49)	0.05
Oxygen						
NBW	358	Ref		85	Ref	
LBW	105	1.19 (0.86–1.65)	0.30	23	1.65 (0.86–3.17)	0.13

Significant values (*p* < 0.05) in bold.

For the whole study period, odds ratios did not change significantly compared to what was found in the baseline alone. Odds of suctioning of LBW infants were lower (adjusted odds ratio [aOR] 0.53, 95% CI 0.34–0.82), and they were higher for ventilation (aOR 1.73, 95% CI 1.24–2.42) after adjustments were made for intervention, gender of the infant and for place of delivery (Table 3).

**Table 3.** Odds ratios (OR) adjusted for sex, place of delivery and study period with 95% confidence intervals for a non-crying LBW baby to receive resuscitative measures compared to a non-crying NBW baby during the Helping Babies Breathe study in Nepal.

	Baseline + intervention, *n *= 1191		
	*n*	Adj. OR	*p-value*
Stimulation			
NBW	575	Ref	
LBW	145	0.93 (0.69–1.25)	0.62
Suction			
NBW	865	Ref	
LBW	215	**0.53** (0.34–0.82)	**< 0.01**
Ventilation			
NBW	167	Ref	
LBW	65	**1.73** (1.24–2.42)	**< 0.01**
Oxygen			
NBW	443	Ref	
LBW	128	1.28 (0.96–1.72)	0.10

Significant values (*p* < 0.05) in bold.

## Discussion

The hypothesis that infants would receive inadequate resuscitation because of LBW was not confirmed. We found that the odds were lower for suctioning of LBW infants, but, as the practice generally is excessively used in this setting, the result rather suggests a more optimal handling of LBW infants. Odds of ventilation were higher in the LBW group, contrary to our hypothesis, as ventilation is the most crucial step to initiate in infants where resuscitation is needed [24]. Results from the regression analyses were similar in both study periods, but only statistically significant in the baseline, reflecting the higher statistical power used in the baseline. However, when analysing both periods together and adjusting for the HBB training, results were in line with the baseline, suggesting that the intervention did not change the resuscitation practice for LBW compared to NBW infants in this setting. The finding of higher odds of ventilation of LBW infants supports the previous finding of a reduced rate of intrapartum stillbirths after training in HBB, as almost half of those stillbirths were LBW infants [8]. The data for gestational age in this study were not complete, but the cases with available information on gestational age confirmed the global estimates of the relatively high proportion of moderately premature infants [25,26]. This study focused on evaluating differences in steps of neonatal resuscitation between LBW and NBW infants; therefore it is difficult to compare with previous observational studies of HBB interventions. However, HBB training in Nepal and India resulted in an increased rate of ventilation whereas the same intervention in Tanzania demonstrated a lower rate of ventilation after training [6–8].

The rate of stimulation of non-breathing infants was reduced after the HBB intervention for the whole study population. One explanation could be that the protocol clearly states the drying of all infants as the first step, followed by a brief stimulation by rubbing the back of the infant if drying does not result in breathing. When observing some of the videos, prolonged and sometimes slightly violent practices of stimulation were present at the facility during the baseline period. As stimulation after the intervention was shorter and health workers moved faster to initiate ventilation, the observers did not note the shorter periods of stimulation as such in the data sheet. The inter-rater reliability of stimulation was also admittedly low in previous studies of the data [16]. Correct tactile stimulation is hard to define, and guidelines only recommend that it should be one of the initial steps, and do not describe in detail how it should be performed [27].

The usage of suctioning of the lower airways was excessive at the studied facility both before and after the intervention for both weight groups. When observing the video recordings we found that, during the baseline period, a catheter was always used, and suctioning of the lower airways was routine when the intervention was applied. After HBB training, some health workers used the penguin suction devices provided by the HBB package, but catheters were still utilised in many cases where suction was performed. The practice of routine lower airway suctioning dates back many decades to when both intrapartum and post-partum suctioning of neonates seemed logical, especially in the presence of meconium-stained fluids, and studies did not confirm any morbidity correlated to such suctioning [28]. This has lately been refuted. If strictly following international guidelines from 2015, routine suction of the lower airways should never be performed, even in cases of depressed infants with meconium-stained fluids, and it should only be considered if there is apparent obstruction of the airway [27]. On the contrary, unnecessary suctioning of the lower airways can possibly harm the infant by causing injury or preventing breathing and reducing the heart rate. Although the practice of excessive suctioning was reduced by the introduction of the HBB protocol for the whole study population, a still-high rate of suctioning prevailed after the training [8]. This emphasises the rigidity of procedure and resistance to change in clinical practice, but might also be explained by the design of the HBB protocol regarding suctioning. In HBB, it is stated that ‘clearing the airway’ is needed if the baby is not crying or breathing well after drying. The section describes only shallow suctioning of the mouth and nose up to 5 cm beyond the lips using a bulb syringe, which is also recommended in international guidelines where ventilation is indicated [27]. However, if deep suctioning of the lower airways has been practised for a long period of time at the facility, such a cautious statement in the protocol might not be enough to change the clinical practice of frequent deep suctioning.

Oxygen was administered to many infants in our study. Proportions were reduced after the intervention as expected as the HBB protocol does not include oxygen administration. Rather, HBB suggests transfer to more advanced care if the initial steps of stimulation, clearing of airway and ventilation are not sufficient. There is no evidence supporting the notion that a correct titration of oxygen will increase survival when neonatal resuscitation is initiated with room air as opposed to 100% oxygen [29]. Rather, oxygen should be titrated according to pre-ductal saturation [30]. As oxygen blenders and measurements of oxygen saturation are rarely available at basic resuscitation stations in low-income facilities, the administration of oxygen should not be a priority.

Our study has some limitations. Firstly, we used infants classified as non-crying from the video material as a proxy to define the need for resuscitation, but we cannot be sure how strong this may be as a predictor. When comparing with low Apgar scores, we found that only a few of the babies defined as crying had an Apgar score below the chosen cut-offs, at both one minute and five minutes. However, many infants who were defined as non-crying in the video material also did not have low Apgar scores. Ideally, an evaluation of whether health workers are performing according to guidelines for a particular intervention would include continuous intrapartum foetal heart rate monitoring and early postnatal electrocardiogram. Secondly, we did not have reliable data on the sequence of interventions during resuscitation. For example, a non-crying baby might have responded to stimulation and therefore there was no need for subsequent ventilation. Thirdly, in the available data, the LBW group contained 10 very, and extremely, premature infants who, in this setting, could be considered to have very low odds of survival, which could have affected the actions taken by the health workers. We chose only birth weight to represent immaturity because of the lack of data on gestational age. Low gestational age combined with LBW could have been a more appropriate representation. For comparison, we calculated ORs for the whole study period, excluding infants with extremely low birth weight (< 1000 grams). The findings were similar and we therefore did not present the results in detail. Fourthly, there could have been a Hawthorne effect in the study as the camera might have influenced the steps taken by the health workers [31]. Finally, regarding infants who were considered too immature, being either extremely premature or SGA, health workers could have decided not to take the infant to the resuscitation table at all, and instead to register the infant as a stillbirth. In such cases, no video recording was available for analysis.

Using video to assess adherence to guidelines in neonatal resuscitation in low-income settings is a promising tool for quality improvement. However, it has its limitations, as demonstrated in this study. In large data-sets, using trained staff when analysing video poses a problem with intra- and inter-rater reliability and it is also very time-consuming. Using better-quality video cameras and the addition of sound could potentially solve some of the problems of gathering reliable data for video interpretation. There is also a need to include coverage of the steps taken between birth and the arrival of the newborn at the resuscitation table. Future possibilities, such as using deep-learning software and allowing for a computer protocol to automatically process the resuscitation videos, should also be explored.

## Conclusion

Low birth weight was associated with higher odds of performing ventilation and lower odds of suctioning when analysing video recordings of non-crying infants resuscitated in a low-income facility setting. Low birth weight does not seem to be a predictor of the decision to withhold resuscitation in this context; however, the video data used did not include decisions taken outside the resuscitation table. The frequent routine use of suctioning of the lower airways is not supported by evidence and needs to be further addressed in resuscitation protocols for low-income settings as its use can cause complications. More robust methods for assessing skills in neonatal resuscitation, including improved video recording and analysis, are needed.

## Paper context

A reduction of intrapartum stillbirth following training in neonatal resuscitation in low-income settings suggests that infants who previously would have been registered as stillbirths are successfully revived. What determines the decision whether to resuscitate is poorly understood. In this study we used video recordings from a facility in Nepal to compare resuscitation of low and normal birth weight infants. Low birth weight was not a predictor of deferred resuscitation in this context.

## Appendix

STROBE Statement – checklist of items that should be included in reports of cohort studies.

**Table:**

	Item No.	Recommendation	Line no. or section in manuscript
**Title and abstract**	1	(*a*) Indicate the study’s design with a commonly used term in the title or the abstract	Title
		(*b*) Provide in the abstract an informative and balanced summary of what was done and what was found	Abstract
		**Introduction**	
Background/rationale	2	Explain the scientific background and rationale for the investigation being reported	Introduction
Objectives	3	State specific objectives, including any prespecified hypotheses	Introduction
		**Methods**	
Study design	4	Present key elements of study design early in the paper	Methods
Setting	5	Describe the setting, locations, and relevant dates, including periods of recruitment, exposure, follow-up, and data collection	Methods No follow-up In the design
Participants	6	(*a*) Give the eligibility criteria, and the sources and methods of selection of participants. Describe methods of follow-up	Methods No follow-up
		(*b*) For matched studies, give matching criteria and number of exposed and unexposed	Not a matched design
Variables	7	Clearly define all outcomes, exposures, predictors, potential confounders, and effect modifiers. Give diagnostic criteria, if applicable	Methods
Data sources/measurement	8*	For each variable of interest, give sources of data and details of methods of assessment (measurement). Describe comparability of assessment methods if there is more than one group	Methods Only birth weight separated the groups
Bias	9	Describe any efforts to address potential sources of bias	Methods
Study size	10	Explain how the study size was arrived at	Available recordings; Methods
Quantitative variables	11	Explain how quantitative variables were handled in the analyses. If applicable, describe which groupings were chosen and why	Groups adjusted for heaping; Methods
Statistical methods	12	(*a*) Describe all statistical methods, including those used to control for confounding	Methods
		(*b*) Describe any methods used to examine subgroups and interactions	Crying and non-crying subgroups; Methods
		(*c*) Explain how missing data were addressed	Only available videos were used and data on birth weight were not missing; Methods
		(*d*) If applicable, explain how loss to follow-up was addressed	No follow-up
		(*e*) Describe any sensitivity analyses	NA
		**Results**	
Participants	13*	(a) Report numbers of individuals at each stage of study – e.g. numbers potentially eligible, examined for eligibility, confirmed eligible, included in the study, completing follow-up, and analysed	Figure 2
		(b) Give reasons for non-participation at each stage	All available included
		(c) Consider use of a flow diagram	Figure 2
Descriptive data	14*	(a) Give characteristics of study participants (e.g. demographic, clinical, social) and information on exposures and potential confounders	Methods
		(b) Indicate number of participants with missing data for each variable of interest	NA
		(c) Summarise follow-up time (e.g. average and total amount)	NA
Outcome data	15*	Report numbers of outcome events or summary measures over time	Figure 2, Tables 2 and 3
Main results	16	(*a*) Give unadjusted estimates and, if applicable, confounder-adjusted estimates and their precision (e.g. 95% confidence interval). Make clear which confounders were adjusted for and why they were included	Tables 2 and 3 and Methods
		(*b*) Report category boundaries when continuous variables were categorized	Methods and Methods
		(*c*) If relevant, consider translating estimates of relative risk into absolute risk for a meaningful time period	NA
Other analyses	17	Report other analyses done – e.g. analyses of subgroups and interactions, and sensitivity analyses	Discussion
		**Discussion**	
Key results	18	Summarise key results with reference to study objectives	Discussion
Limitations	19	Discuss limitations of the study, taking into account sources of potential bias or imprecision. Discuss both direction and magnitude of any potential bias	Discussion
Interpretation	20	Give a cautious overall interpretation of results considering objectives, limitations, multiplicity of analyses, results from similar studies, and other relevant evidence	Conclusion
Generalisability	21	Discuss the generalisability (external validity) of the study results	Discussion
		**Other information**	
Funding	22	Give the source of funding and the role of the funders for the present study and, if applicable, for the original study on which the present article is based	Funding

Notes: *Give information separately for exposed and unexposed groups.

An Explanation and Elaboration article discusses each checklist item and gives methodological background and published examples of transparent reporting. The STROBE checklist is best used in conjunction with this article (freely available on the websites of PLoS Medicine at http://www.plosmedicine.org/, Annals of Internal Medicine at http://www.annals.org/, and Epidemiology at http://www.epidem.com/). Information on the STROBE Initiative is available at http://www.strobe-statement.org.
